# Decreased expression of p53 is associated with down expression of zyxin in breast cancer

**DOI:** 10.1002/hsr2.2288

**Published:** 2024-08-01

**Authors:** Rezvan Rostampour, Kiana Bahremand, Hossein Mohammadi, Seyed Askar Roghani, Ebrahim Shakiba, Mohammad Taghi Goodarzi, Soheila Asadi

**Affiliations:** ^1^ Department of Clinical Biochemistry Kermanshah University of Medical Sciences Kermanshah Iran; ^2^ Nano Drug Delivery Research Center, Health Technology Institute Kermanshah University of Medical Sciences Kermanshah Iran; ^3^ Clinical Research Development Center, Imam Reza Hospital Kermanshah University of Medical Science Kermanshah Iran; ^4^ Department of Biochemistry Shahrood Branch, Islamic Azad University Shahrood Iran

**Keywords:** breast cancer, p53, zyxin

## Abstract

**Background and Aims:**

Breast cancer (BC) is considered one of the most common malignant tumors leading to death in women, and genetic factors have a crucial role in BC pathogenesis. Zyxin (ZYX) is one of these factors that may be important in p53 level and function. Thus, the present work aimed to investigate the ZYX gene and protein expression in tumor tissue and matched margin tissue and its correlation with the p53 expression.

**Methods:**

In a present case‐control study, 30 tumors and 30 matched margin tissues were obtained from Iran Tumor Bank/Tehran University of Medical Sciences. Real‐time polymerase chain reaction and western blot analysis techniques were applied to evaluate the genes and protein expression, respectively.

**Results:**

The data showed that expression of the ZYX gene in tumor tissues significantly decreased (*p* = 0.0274) compared to matched margin tissues. In contrast, the p53 gene expression in tumor tissues had no significant difference with matched margin tissues. Additionally, we observed that ZYX and p53 genes expression in tumor tissues of estrogen receptor‐positive patients had significant elevation than estrogen receptor‐negative patients (*p* < 0.001, *p* < 0.001, respectively). The data of the western blot analysis technique showed that protein expression of ZYX (*p* = 0.0024) and P53 protein (*p* = 0.0218) in tumor tissues was significantly reduced compared to matched margin tissues. Additionally, our analysis showed a direct and significant correlation between the expression of ZYX and p53 proteins (*r* = 0.7797, *p* = 0.0126) and expression of ZYX and p53 genes (*r *= 0.3079, *p* = 0.0187).

**Conclusion:**

Based on our observation, ZYX might have a tumor suppressor role and is associated with p53.

## 1 INTRODUCTION

Breast cancer (BC) is one of the most common malignant tumors, and its incidence is increasing every year. In 2019, approximately 316,700 new cases of BC were confirmed in US women; the growth rate is estimated at approximately 0.3% per year.^1^ It is predicted that by 2050, there will be about 3.2 million new BC cases worldwide each year.^1^ Despite the use of various types of treatment methods in BC, including endocrine therapy, systemic chemotherapy, and new therapeutic approaches, the outcome of some types of metastatic BC has not been favorable; thus, more investigations are needed to improve the treatment. Genetic factors have crucial roles in the pathogenesis of BC, and one of the essential factors that might be involved in the pathogenesis of BC is zyxin (ZYX).^2^, ^3^ The ZYX, as a phosphoprotein with 572 amino acids, has an N‐terminal (containing proline‐rich sequences) and LIM at the C‐terminal, and the ZYX, by these domains can interact with various proteins. This protein mainly focuses on focal adhesions (FAs) and affects actin cytoskeletal dynamics, cell motility, and signal transduction.^4^ According to the literature, ZYX has various roles in cancer pathogenesis depending on the type of organ involved, test conditions, and the presence of some proteins that ZYX interacts with them.^5^, ^6^ Some studies indicated that ZYX may be associated with homeodomain interacting protein kinase2 (HIPK2) through the LIM domain and acts as an essential regulator of cell death due to DNA damage by regulating the HIPK2‐p53 signaling axis.^7^ The p53 has an important function in cell cycle arrest regulation, DNA repair, apoptosis, autophagy, and metabolism.^8^ Based on the above information, we hypothesized that ZYX might be associated with p53; therefore, we aimed to compare the expression levels of the ZYX gene and protein in tumor tissue and tumor margin and its possible coronation with the protein expression of p53.

## 2 MATERIAL AND METHODS

### 2.1 Sample collection

In a present case‐control study, 30 samples of BC tissue as case and 30 tumor margin tissue as control were obtained from the Iran National Tumor Bank, which was founded by the Cancer Institute of Tehran University of Medical Sciences for Cancer Research (Iran), from Iran Tumor Bank/Tehran University of Medical Sciences. The patients with chemotherapy, patients with a history of radiotherapy or taking anti‐inflammatory drugs, and patients with BC due to metastasis of another type of cancers were excluded from the study.

### 2.2 Determination of *ZYX* and p53 gene expression by real‐time PCR

The total RNA from BC tissues (fresh‐frozen) and matched tumor margin tissues (fresh‐frozen) were extracted using RNX‐Plus solution. Briefly, up to 50 mg of fresh‐frozen tissue was homogenized using liquid nitrogen. Then 1 mL of RNX‐Plus solution was added and the tube was incubated 5 min at room temperature. After adding 200 µL of chloroform, the tube was incubated 5 min on ice and then centrifuged at 12,000 rpm at 4°C for 15 min. The aqueous phase was transferred to new tube, and equal volume of isopropanol was added and the tube was incubated at 4°C for 40 min. In the next step, the tube was centrifuged at 12,000 rpm at 4°C for 15 min, then, the supernatant was discarded and 1 mL of 75% ethanol was added to the pellet. Finally, the tube was centrifuged at 4°C for 8 min at 7500 rpm, and the pellet was dissolved in RNAase and DNAase‐free water. The RNA quantity and integrity were tested by Nanodrop spectrophotometer (Thermo Scientific™ NanoDrop™ One Microvolume UV‐Vis Spectrophotometers, Thermo Fisher Company) and 1% tris acetate EDTA‐agarose electrophoresis. The complementary DNA (cDNA) was synthesized using cDNA synthesis kit (Yekta Tajhiz Azma) based on manufacturing company. The list of primers used in this study is presented in Table 1, and the β actin gene was used as a housekeeping gene. Real‐time polymerase chain reaction (PCR) amplification was performed via SRealQ Plus 2x Master Mix Green kit (AMPLIQON) in Light Cycler 480® (Roche). The data of RT‐PCR were normalized with β actin (housekeeping gene), and 2^−ΔΔ*C*^T was used for calculating the fold change of *ZYX* and p53.

**Table 1:** The primer sequences used in real‐time polymerase chain reaction.

Genes		Primer sequence
β actin	F	CATGTACGTTGCTATCCAGGC
	R	CTCCTTAATGTCACGCACGAT
ZYX	F	ATCCTCAGAGGCAGAATGTGG
	R	AAGCAGGCGATGTGGAAC
p53	F	CAGCACATGACGGAGGTTGT
	R	TCATCCAAATACTCCACACGC

### 2.3 Determination of ZYX and p53 proteins level by western blot analysis

In the first step, for extracting total protein from fresh frozen tissues, the radioimmunoprecipitation assay buffer and protease inhibitor cocktail (Kiazist) were added to crushed tissues and incubated on ice for 15 min. Then, the tissues homogenate were centrifuged at 20,000 rpm and 4°C for 20 min. The supernatant was aliquoted in preferred volumes and stored at −20°C. For assessing total protein levels in tissue extracts, the bicinchoninic acid assay method (KIAZIST: KBCA‐96. IR) was used according to the manufacturer's company.

For the western blot analysis assay, in the first step, the Laemmli‐SDS‐PAGE^9^ was used to separate protein using 40 μg of total protein. Subsequently, protein bands were transferred from the gel to the nitrocellulose membrane. To block nonspecific sites, the membrane containing the protein bands was incubated with blocking buffer (5% nonfat skim milk; Sigma‐Aldrich) at room temperature for 2 h. After two times washing with phosphate buffered saline containing 0.05% v/v tween 20 (PBST) and one time washing with phosphate‐buffered saline (PBS), the membranes were incubated with primary antibodies, including mouse anti‐ZYX antibody (1:1000 dilution; Sigma‐Aldrich, Z0377), mouse anti‐p53 antibody (1:2000 dilution; STJ, STJ96954), and mouse beta‐actin antibody (1:1000 dilution; Santa Cruz sc‐47778) at 4°C overnight. The membrane was then washed twice with PBST buffer and one time with PBS buffer, each time for 10 min, and subsequently, the membranes were incubated for an hour with a secondary antibody conjugated with horseradish peroxidase (1:3000 dilution; Biolegend). Finally, the membranes were washed three times with PBST buffer each time for 10 min and one time with PBS buffer for 15 min. BioRad enhanced chemiluminescence select western blot analysis detection Reagent (BioRad) was used for visualizing protein bands, and protein bands were scanned densitometrically using ImageJ software (Java 1.6.0.20; National Institute of Health).

### 2.4 Statistical analysis

For statistical analysis of data, version 16 of SPSS software was applied in the present study. All data were presented based on the mean ± standard deviation, and the significance level of statistical tests for all tests was considered *p* < 0.05. The two‐tailed Student's test was applied to analyze quantitative data; the *χ* ^2^ test was used for evaluating qualitative data, and the Pearson correlation coefficient was applied to examine the association between ZYX protein and p53 protein expression.

## 3 RESULTS

### 3.1 Demographic and clinicopathological characteristics of the patients

Demographic information of the patients, including age, sex, and method of access to tissue in patients, were listed in Table 2. Additionally, the clinicopathological characteristics, including tumor size, histology, tumor grading, vascular and lymphatic invasion, tumor staging, and tumor node metastasis staging, were listed in Table 3.

**Table 2:** Demographic information of the patients.

Characteristic	Categorization	*N* (%)
Age	49.13 ± 11.99	—
Sex	Female	30 (100)
	Male	0 (0)
Type of procedure	Surgical resection	30 (100)
Site of primary	Left breast	14 (46.7)
	Right breast	15 (50)
	Bilateral	1 (3.3)

**Table 3:** Clinicopathological characteristics of the patients.

Characteristic	Categorization	*N* (%)
Tumor size	8.25 ± 7.96	—
Histology	Infiltrating ductal, NOS	1 (3.3)
	Infiltrating lobular, NOS	2 (6.7)
	Invasive ductal carcinoma	22 (73.3)
	Others	4 (13.3)
	Infiltrating ductal, mucinous (colloid)‐invasive ductal carcinoma	1 (3.3)
Histology grade	Grade I (well differentiated)	2 (6.7)
	Grade II (intermediate‐moderately differentiated)	14 (46.7)
	Grade III (high‐poor differentiated)	10 (33.3)
	Grade X (unknown)	4 (13.3)
Necrosis presence	Yes	15 (50)
	No	15 (50)
Vascular invasion	Yes	19 (63.3)
	No	11 (36.7)
Pathological T	T1, NOS	1 (3.3)
	T1a	1 (3.3)
	T2	16 (53.3)
	T3	4 (13.3)
	T4, NOS	2 (6.7)
	T4a	3 (10)
	T4b	3 (10)
Pathological N	N0 (ITC)	1 (3.3)
	N0, NOS	15 (50)
	N1, NOS	6 (20)
	N1a	2 (6.7)
	N2, NOS	2 (6.7)
	N2a	2 (6.7)
	N3, NOS	1 (3.3)
	N3a	1 (3.3)
Clinical metastasis	M0	29 (96.7)
	M1	1 (3.3)
TNM	Stage IIA	9 (30)
	Stage IIB	9 (30)
	Stage IIIA	4 (13.3)
	Stage IIIB	5 (16.7)
	Stage IIIC	2 (6.7)
	Stage IV	1 (3.3)
Estrogen receptor	Positive NOS	9 (30)
	Negative	9 (30)
	Missing	12 (40)
Progesterone receptor	Positive NOS	9 (30)
	Negative	8 (26.7)
	Missing	13 (43.3)
Her‐2 receptor	Positive (1–2)	2 (6.7)
	Positive (3–4)	2 (6.7)
	Negative	11 (36.7)
	Mossing	15 (50)

Abbreviation: TNM, tumor node metastasis.

### 3.2 Expression of *ZYX* and p53 genes by 2^−∆∆*C*^T method

**Figure 1:**
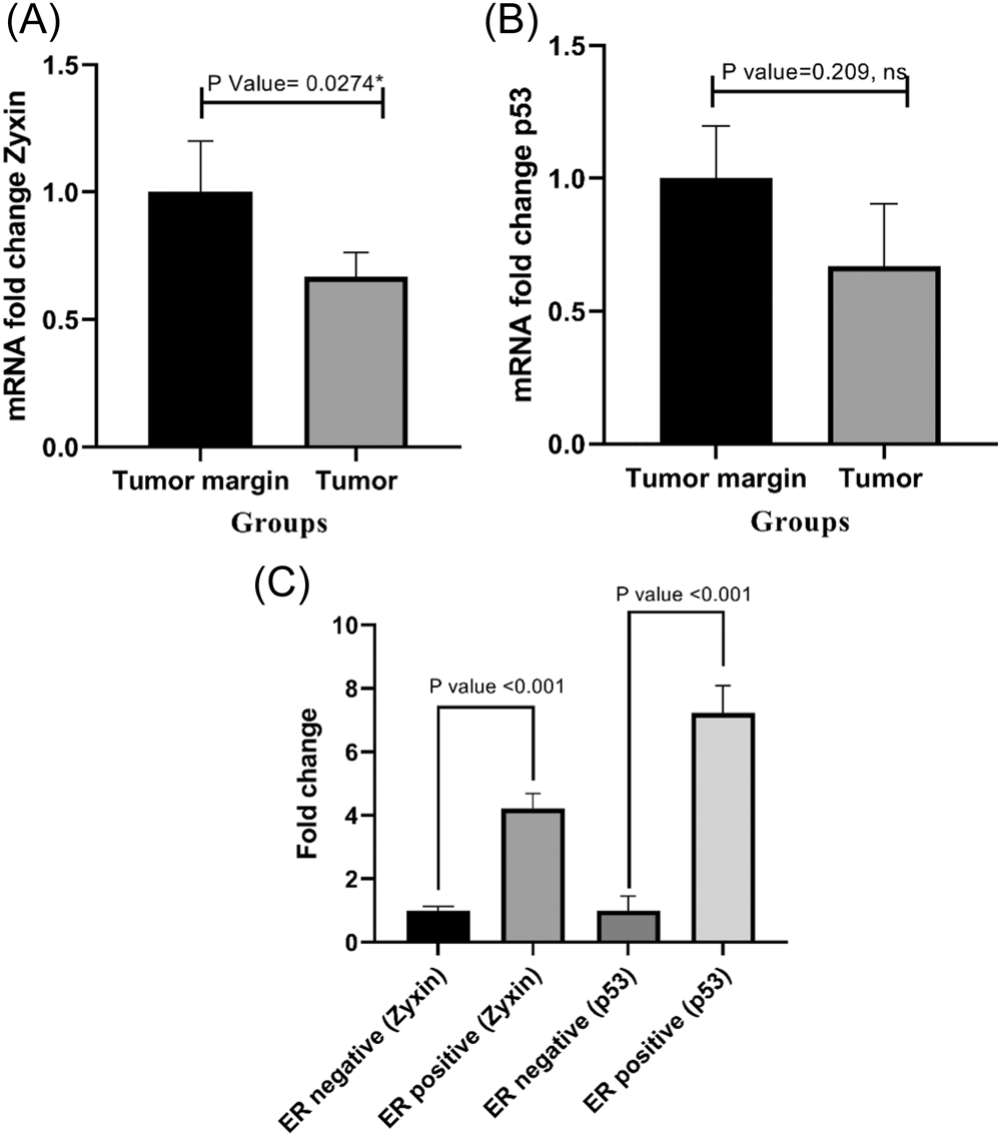
Expression of *ZYX* (A), p53 (B) genes in BC patients' tumor tissues (*n* = 30) and tumor margin tissues (*n* = 30), ZYX and p53 genes expression in tumor tissues of estrogen receptor‐positive (*n* = 9) and estrogen receptor‐negative patients (*n* = 8). (C) Data are presented as mean ± standard deviation; the *p* value < 0.05 is considered as significant. BC, breast cancer; ER, estrogen receptor; mRNA, messenger RNA.

As presented in Figure 1A, the expression of the *ZYX* gene in tumor tissues significantly decreased (*p* = 0.0274) compared to matched margin tissues. At the same time, our observations indicated no significant difference in p53 gene expression between tumor tissues and matched margin tissues (Figure 1B). Furthermore, we assessed the expression of ZYX and p53 in tumor tissues of estrogen receptor‐positive and estrogen receptor‐negative patients. Our finding indicated that the expression of ZYX and p53 genes in tumor tissues of estrogen receptor‐positive patients had significant difference from estrogen receptor‐negative patients (*p* < 0.001, *p* < 0.001, respectively) (Figure 1C).

### 3.3 Results of western blot analysis analysis

**Figure 2:**
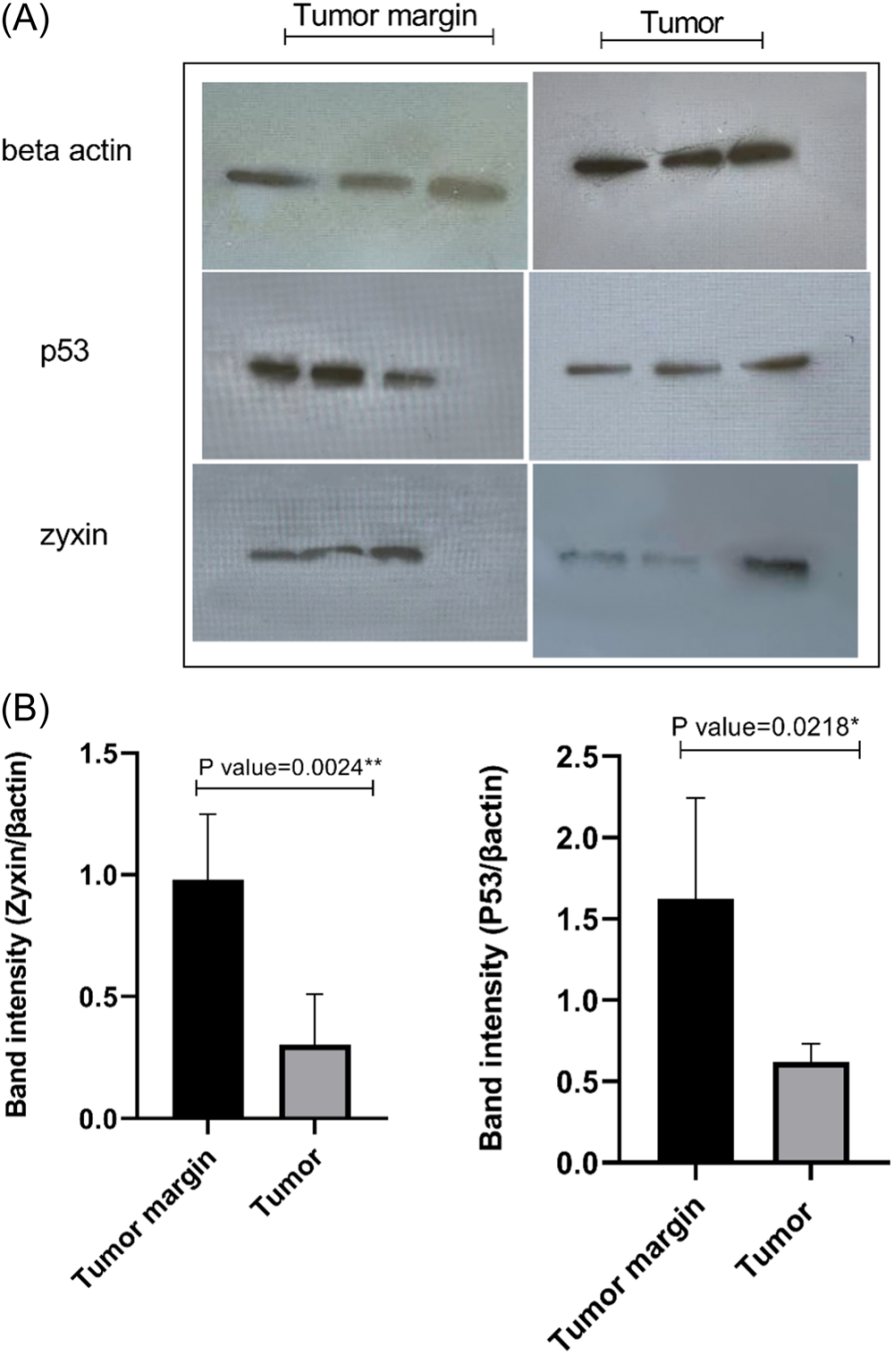
Expression of ZYX and p53 proteins in breast tumor tissues (*n* = 6) and tumor margin tissues (*n* = 6) (six tissues from each group were randomly selected). (A) Western blot bands of ZYX and p53 proteins. (B) Expression of ZYX and p53 proteins relative to β actin. Data are presented as mean ± standard deviation; the *p* value < 0.05 is considered as significant.

Our findings indicated that the expression of ZYX and P53 proteins in tumor tissues decreased compared to matched margin tissues (Figure 2A). Additionally, our assessment revealed that protein levels of ZYX (*p* = 0.0024) and P53 (*p* = 0.0218) in tumor tissues significantly decreased compared to matched margin tissues (Figure 2B).

### 3.4 Correlation analysis of ZYX and p53 protein expression

A direct and significant correlation was observed between ZYX and P53 protein expression (*r* = 0.7797, *p* = 0.0126). Additionally, our analysis revealed direct and significant correlation between ZYX and p53 genes expression (*r* = 0.3079, *p* = 0.0187).

## 4 DISCUSSION

The present case‐control study was performed to evaluate the ZYX gene and protein expression and its association with p53 expression. Based on our observation, expression of the ZYX gene in tumor tissue was reduced compared to matched margin tissue. However, there was no significant difference in the p53 gene expression between tumor and matched margin tissue. Interestingly when we compared the expression of p53 and ZYX in estrogen receptor‐positive compared to estrogen receptor‐negative patients, we found that estrogen receptor‐positive patients had higher expression of p53 than estrogen receptor‐negative patients. Regarding protein expression, the levels of Zyx and p53 proteins in tumor tissue decreased compared to the matched margin tissues.

The p53 gene inactivation is crucial in developing many types of cancers. In stress conditions, the p53 mediates cell cycle arrest, DNA repair, apoptosis, and autophagy; furthermore, p53 activity is negatively regulated by MDM2.^10^ Stefano et al. showed that HIPK2 increases total P53 transcriptional activity by overcoming MDM2 inhibition and induces P53 functions such as apoptosis.^11^ On the other hand, ZYX is a crucial regulator for HIPK2, and it is observed that reduction of ZYX level leads to degradation of HIPK2; therefore, ZYX might be considered as the primary regulator of DNA‐induced cell death by regulating the HIPK2‐p53 signaling pathway.^7^

However, the study by Zhou et al. suggested that ZYX probably promotes cancer growth and proliferation.^12^ The contradictory function of this protein may be related to the organ involved, test conditions, and interaction with other proteins and factors. In another study, it was shown that ZYX is associated with cell proliferation.^13^ Another study that was conducted by Grunewald et al. showed an oncogenic function for ZYX in BC; although, their study was performed on cell line and their results showed that transfection of cell line with siRNA had no effect on ZYX protein expression but changed ZYX localization.^14^

It was shown that ZYX interacts directly with myopodin (Synaptopodin 2), a tumor suppressor gene, which reported that this protein is deleted in patients with prostate cancer. Thus, ZYX might be necessary in tumor suppressor function of myopodin.^15^ The study of YU et al. found that deletion of the sequence required for myopodin‐ZYX interaction could affect the suppression of cell motility and invasion mediated by myopodin.^5^ ZYX interaction with myopodin has also been reported in testicular tumors, which also play a role in suppressor tumors due to this type of interaction. A study at the Memorial Sloan Kettering Cancer Center found that myopodin potentially suppresses tumors, and this protein interacts with ZYX.^16^

ZYX can exert its influence through signaling pathways. To illustrate, in the evaluation of 173 subjects with transitional cell carcinoma of the bladder, it was reported that low levels of ZYX had a significant association with tumor grade and stage.^17^ This role of ZYX might be associated with the β‐catenin signaling pathway.^18^

According to previous studies and the result of our study, it can be concluded that ZYX has both oncogenic and tumor‐suppressant effects, and its function is associated with the organ involved and its mutual protein partners. Most of the effects of ZYX are due to its interactions with other proteins, and in general, ZYX alone does not have an independent effect. Our study investigated the ZYX and p53 genes and protein expression as one of the most significant markers of the suppressive tumor. Our observation showed a significant correlation between ZYX and P53 proteins. Therefore, based on our results, ZYX may have a tumor suppressor function in BC.

### 4.1 Study limitation

1. Like other observational studies, our study had limitations that affected the accuracy and bias of the study results. Since our selected samples were selected from only one sex, the results of a study performed solely on female breast tissue could not be generalized to BC in men.
2. Since BC is affected by race, geographical area, drug treatments, culture, eating habits, occupation, and so on,^19^ and our selected samples were only from a limited area, our findings cannot be attributed to all races and ethnicities and different geographical areas.
3. Because the genes are highly influenced by test conditions and existing equipment,^20^ this can affect the results obtained under dissimilar conditions. On the other hand, genes do not act independently in vivo. They are affected by associated proteins, comorbidities, and different metabolic pathways, so under the effects of these factors, they can exhibit various behaviors.
4. The patient's survival information and their correlation with ZYX expression could provide useful information. However, the samples of present study were received from Iran National Tumor Bank and the patients from different cities of Iran were referred to this center; thus, it was not possible for us to follow up the patients.
5. If it was possible to set in vitro study using the samples of participants in the present study and investigate the effect of treatment of cells with MDM2 or USP7 inhibitors on ZYX expression, the results would provide important information about ZYX; but because fresh‐frozen tissues were used in present study so the investigation of this hypothesis was not possible. The results of an experimental study under controlled conditions and investigation of limited factors cannot be conclusive.

## 5 CONCLUSION

Based on our findings, ZYX might have a tumor suppressor role and is associated with p53.

## AUTHOR CONTRIBUTIONS

**Rezvan Rostampour**: Investigation; methodology; writing—original draft; software. **Kiana Bahremand**: Methodology; investigation; writing—original draft; validation; writing—review and editing. **Hossein Mohammadi**: Methodology; investigation; writing—review and editing. **Seyed Askar Roghani**: Investigation; methodology; writing—review and editing. **Ebrahim Shakiba**: Investigation. **Mohammad Taghi Goodarzi**: Writing—review and editing; validation; methodology. **Soheila Asadi**: Conceptualization; investigation; writing—original draft; methodology; validation; visualization; writing—review and editing; software; formal analysis; project administration; data curation; supervision.

## CONFLICT OF INTEREST STATEMENT

The authors declare no conflict of interest.

## ETHICS STATEMENT

The present study was approved by the ethics committee of Kermanshah University of Medical Science (IR.KUMS.REC.1400.158). All participants had informed consent for participating in present work and after agreed to participate in the present study, their primary information were collected and recorded.

## TRANSPARENCY STATEMENT

The lead author Soheila Asadi affirms that this manuscript is an honest, accurate, and transparent account of the study being reported; that no important aspects of the study have been omitted; and that any discrepancies from the study as planned (and, if relevant, registered) have been explained.

## Data Availability

All analyzed data will be available on reasonable request from the corresponding authors.
